# Potential effects of peripheral neuropathy on brain function in patients with type 2 diabetes mellitus

**DOI:** 10.3389/fendo.2024.1448225

**Published:** 2024-11-11

**Authors:** Dongsheng Zhang, Yang Huang, Xiaoling Zhang, Wanting Liu, Yitong Guan, Jie Gao, Xiaoyan Lei, Min Tang, Kai Ai, Xuejiao Yan

**Affiliations:** ^1^ Department of MRI, Shaanxi Provincial People’s Hospital, Xi’an, China; ^2^ Department of Clinical Science, Philips Healthcare, Xi’an, China

**Keywords:** diabetic peripheral neuropathy, functional connectivity, resting-state fMRI, sensorimotor, type 2 diabetes mellitus

## Abstract

**Background:**

The mechanisms associated between diabetic peripheral neuropathy (DPN) and various brain function abnormalities in patients remains unclear. This study attempted to indirectly evaluate the effect of DPN on brain function in patients with type 2 diabetes mellitus (T2DM) by characterizing the resting-state functional connectivity (FC) of the lower limb sensorimotor cortex (LSM).

**Methods:**

Forty-four T2DM patients with diabetic peripheral neuropathy (DPN), 39 T2DM patients without diabetic peripheral neuropathy (ND), and 43 healthy controls (HCs) underwent a neuropsychological assessment and resting-state functional magnetic resonance imaging examinations to examine the differences in FC between the LSM and the whole brain. The relationships of FC with clinical/cognitive variables were examined.

**Results:**

In comparison with the HCs group, the ND group showed reduced FC of the LSM with the right lateral occipitotemporal cortex (LOTC) and increased FC with the medial superior frontal gyrus (SFGmed), while the DPN group showed reduced FC of the LSM with the right cerebellar lobule VI, the right LOTC, the rostral prefrontal cortex (rPFC), and the anterior cingulate gyrus (ACC). Moreover, in comparison with the ND group, the DPN group showed reduced FC of the LSM with the ACC, SFGmed, and rPFC. In the DPN group, the FC between the LSM and right cerebellar lobule VI was significantly correlated with fasting blood glucose levels (r = -0.490, *p* = 0.001), and that between the LSM and ACC was significantly correlated with the Montreal Cognitive Assessment score (r = 0.479, *p* = 0.001).

**Conclusions:**

Patients with T2DM may show abnormal motion-related visual perceptual function before the appearance of DPN. Importantly, DPN can influence the brain regions that maintain motion and motor control, and this effect is not limited to motor function, which may be the central neuropathological basis for diabetic peripheral neuropathy.

## Introduction

Diabetic peripheral neuropathy (DPN) is the most common complication of type 2 diabetes mellitus (T2DM), presenting in more than 50% of patient (1). DPN is a key initiating factor in the development of diabetic foot ulcers and the most common cause of non-traumatic lower limb amputations in most high-income countries (2). Unfortunately, no easy-to-use biomarkers are currently available for early diagnosis of DPN (3). The pathogenesis underlying DPN includes peripheral sensitization and central sensitization mechanisms (4). A large body of evidence from recent studies suggests that alterations in the central nervous system (CNS) is involved in the pathogenesis of DPN (5–7). Therefore, exploring the characteristics of altered brain function in patients with DPN may provide ideas for understanding its pathogenesis and developing more targeted diagnostic and therapeutic programs.

The central vulnerable brain regions in patients with DPN are mainly located in the primary sensorimotor cortex (SM), and structural and functional abnormalities in this region are associated with the severity of peripheral neuropathy (5) (8–11),. In addition to abnormalities in the motor cortex, patients with DPN show reduced gray matter volume in the deep gray matter nuclei and dorsolateral prefrontal cortex (9), and thinning of the cingulate, insula, and prefrontal cortex cerebral cortex (10). Other MRI studies (12, 13) have shown that in addition to impaired central motor and somatosensory pathways, these patients also show abnormal alterations in vision and many cognition-related brain regions. However, previous studies did not clarify whether the abnormal changes in other brain regions are associated with sensory neuropathy in patients with DPN. The postural instability and high gait variability in patients with DPN can increase the likelihood of falls (14, 15). Postural control requires the co-coordination of the vestibular, visual, and somatosensory systems (16), which also seems to suggest that sensory neuropathy may produce a range of alterations to the CNS. On the other hand, multiple studies (17, 18) have suggested that in addition to showing reduced physical balance, patients without DPN also show subtle but obvious degeneration of all three sensory systems (somatosensory, visual, and vestibular). Although a previous study indicated that neuronal activity in the sensorimotor cortex of patients without DPN did not show significant abnormal changes (12), the possibility of abnormal functional coupling of the sensorimotor cortex with other brain regions before the emergence of DPN in patients with T2DM needs to be further explored.

Bilateral lower-extremity sensory neuropathy is the first and main symptom of DPN. Sensory information from the lower limbs is transmitted to the spinal cord via somatosensory fibers, and then to the SM via the spino-thalamic-cortical and medial lemniscus-thalamic-cortical pathways. Therefore, characterizing the coupling of the sensorimotor cortex mapped by the bilateral lower limbs to whole-brain function may reveal the effects of DPN on brain function in patients. Recently, Fan et al. (19) used connectivity-based parcellation to create a new human brain atlas and defined the lower limb sensorimotor cortex (LSM). This approach allowed researchers to identify functionally meaningful brain regions without asking participants to perform specific tasks (20, 21), and has been widely used in resting-state functional MRI (rs-fMRI) studies (22, 23).

Therefore, this study aimed to investigate the characteristics of the resting-state functional connectivity (FC) between the LSM and the whole brain in T2DM patients with and without DPN, thereby indirectly reflecting the effects of DPN on the patient’s CNS. We hypothesize that even patients without peripheral neuropathy (ND) may show disordered FC with other sensory cortices, and DPN may have extensive effects on the motor and cognitive-related brain areas in the CNS. This study will help reveal the association of peripheral nerve abnormalities with the CNS in patients with T2DM and provide further insight into the pathogenesis of DPN.

## Materials and methods

### Participants

In this study, 86 patients with T2DM were recruited at the Endocrinology Department of Shaanxi Provincial People’s Hospital from May 2020 to October 2022, including 46 DPN and 40 ND patients, while 43 healthy controls (HCs) matched for age, sex, and education level were recruited at the health examination center of our hospital during the same period. All participants were aged 45-70 years, right-handed, and had at least 6 years of education. The Mini-Mental State Examination (MMSE) score of the participants was ≥24. The inclusion criteria for the HC group were as follows: no symptoms of T2DM; fasting blood glucose (FBG) concentration of <6.1 mmol/L; and glycated hemoglobin (HbA1c) level of <6.0%. T2DM was diagnosed in accordance with the 2014 American Diabetes Association diagnostic criteria. Patients without any kind of microvascular complications were assigned to the ND group. T2DM patients who met the following criteria (24) were regarded as having DPN: 1) having a clear history of diabetes; 2) clinical evidence of peripheral neuropathy at or after diagnosis diabetes;Ihaving clinical symptoms of neuropathy (such as pain, numbness, sensory abnormalities), and abnormalities in any of the five tests (ankle reflexes, vibratory sensation, pressure sensation, temperature sensation, and pinprick pain sensation) can be diagnosed;II without clinical symptoms, any two abnormalities in the five tests can also be diagnosed; 3) abnormal results in nerve conduction studies (decreased tibial nerve or common peroneal nerve conduction velocity on electromyography); 4) rule out neuropathy from other causes. Patients with T2DM were receiving stable therapy (diet, oral medications, and/or insulin).

The exclusion criteria for all participants were as follows: severe claustrophobia or contraindications to magnetic resonance imaging (MRI); Parkinson disease, alcoholism, major depression, epilepsy, brain injury, or other neurological or psychiatric disorders; or any other systemic disease. In addition, DPN patients with other complications of diabetes (diabetic nephropathy and retinopathy) and HCs with Montreal Cognitive Assessment (MoCA) scores ≤ 26 were also excluded.

The subjects’ MRI scan times and procedures were performed as previously described (25). This study was approved by the ethics committee of Shaanxi Provincial People’s Hospital, and all participants provided written informed consent. All procedures were performed in accordance with the relevant guidelines and regulations of the Declaration of Helsinki.

### Clinical data collection and neuropsychological tests

The medical history and clinical data of the participants, including their blood pressure level, height, weight, and body mass index (BMI), were obtained from medical records and questionnaires. In addition, the HbA1c, FBG, postprandial blood glucose (PBG), triglyceride (TG), total cholesterol (TC), low-density and high-density lipoprotein cholesterol (LDL/HDL-C), serum urea, and serum creatinine levels were measured by standard laboratory testing. A series of neuropsychological tests were used to evaluate the participants’ mental status and cognitive condition. The MMSE and MoCA were used to assess general cognitive function; the Color Trails Test part 1 and part 2 (CTT-1 and CTT-2) were used to test attention and executive functions (26); the Clock-Drawing Test (CDT) was used to evaluate visuospatial skills (27); the total immediate recall and delayed recall scores in the Rey Auditory Verbal Learning Test (RAVLT) were obtained for assessing memory function (28); and processing speed/attention were evaluated using the Symbol Digit Modalities Test (SDMT) (29). All neuropsychological tests were administered by a psychiatrist with at least 5 years of experience.

### MRI data acquisition

The MRI scans were acquired using a 3.0T MR scanner (Ingenia, Philips Healthcare, The Netherlands) with a 16-channel phased array head coil. Routine T_2_-weighted and fluid-attenuated inversion recovery (FLAIR) sequences were used to exclude visible brain lesions. Sagittal 3-dimensional T_1_-weighted imaging (T_1_WI) was performed using a fast spoiled gradient-echo sequence with the following parameters: repetition time (TR) = 7.5 ms, echo time (TE) = 3.5 ms, flip angle (FA) = 8°, field of view (FOV) = 250 mm × 250 mm, matrix = 256 × 256, slice thickness = 0.55 mm (no gap), and number of slices = 328. The scanning range covered the whole brain. The rs-fMRI images were obtained using a gradient-echo planar sequence with the following parameters: TR = 2,000 ms, TE = 30 ms, number of slices = 34, slice thickness = 4 mm (no gap), FA = 90°, FOV = 230 mm × 230 mm, and matrix = 128 × 128, and number of volumes acquired in each scan = 200. All participants were instructed to close their eyes but stay awake throughout the scan.

Lacunar infarcts and white-matter hyperintensity (WMH) on FLAIR images were quantified using an age-related white-matter change scale (30) with a single-blind method, and participants with ratings >2 were excluded.

### Processing of rs-fMRI data

The rs-fMRI data were preprocessed using the DPABI (http://rfmri.org/dpabi/) software package with the following steps: 1) The first ten time points were discarded to ensure the stability of the magnetic field; 2) slice-timing correction was performed for interleaved acquisitions to correct the time delay between slices; 3) realignment of head motion correction was performed and patients with large head motion (head motion > 1.5 mm and/or translation > 1.5° of rotation in any direction) were excluded; 4) the images were spatially normalized to the standard Montreal Neurological Institute (MNI) space using the EPI template (resampling voxel size = 3 mm × 3 mm × 3 mm); 5) the images were smoothed using a Gaussian kernel with a 6-mm full width at half maximum (FWHM); and 6) multiple regression models involving 24 motion parameters, cerebrospinal fluid signals, white-matter signals, linear trend signals, and the “scrubbing” method were used to reduce the effect of high head motion, and the framewise displacement (FD) threshold was 0.2 mm (31). Finally, a temporal-band filter (0.01–0.08 Hz) was used to regress the effect of physiological noise.

### Functional connectivity analysis

**Figure 1:**
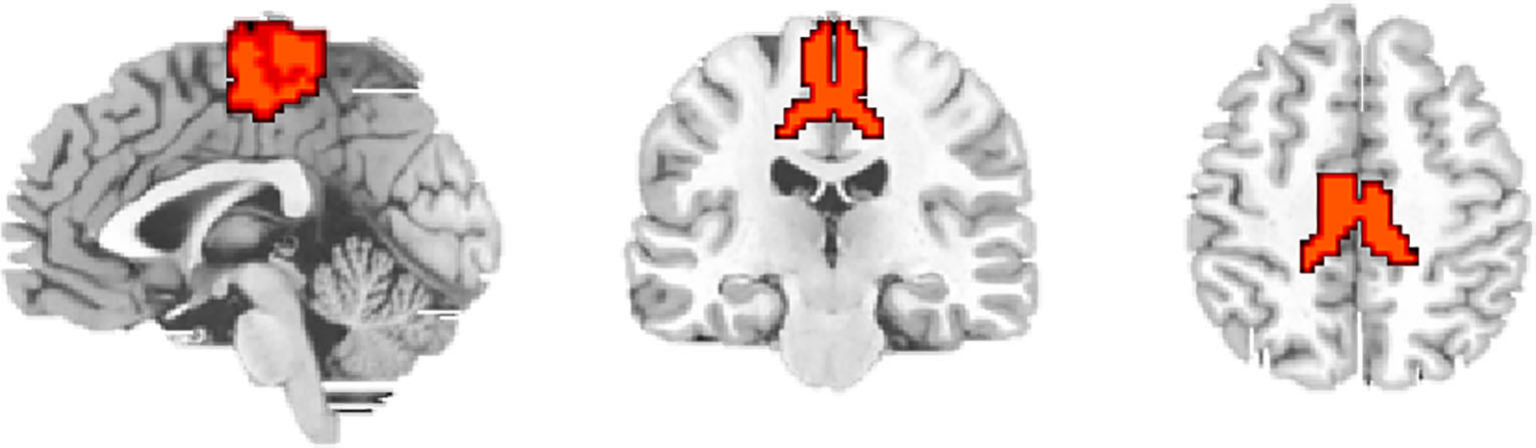
Regions of interest in the lower limb sensorimotor cortex (LSM).

The LSM was defined as the region of interest (ROI) according to the Human Brainnetome Atlas (19). The ROI (**Figure 1**) included PCL_2_1 (Area 1/2/3) and PCL_2_2 (Area 4), which was extracted for voxel-wise FC analyses. For each participant, the mean time series were extracted for ROI, and correlations were calculated between ROI and every other voxel within the brain to obtain FC maps. These FC maps were converted to z-score maps using Fisher’s z transformation to improve normality.

### Statistical analyses

The chi-square test (*χ* ^2^) was used to assess sex-related group differences. One-way analysis of variance (ANOVA) was used to analyze other demographic and clinical and cognitive scores. A two-sample t-test was used to compare disease duration between the DPN and ND groups. These analyzes were performed using SPSS version 24 (IBM Corporation, Armonk, NY, United States), and *p* < 0.05 was considered to be statistically significant.

Statistical analyses of seed-based FC were performed using DPABI software. Analysis of covariance (ANCOVA) was performed to investigate the regions showing significant differences in FC among the three groups, with age, sex, and years of education as covariates, the results corrected for multiple comparisons using the Gaussian random field (GRF) method to minimize type I error (32) (GRF-corrected *p* < 0.001, cluster level *p* < 0.05, cluster > 50). The ANCOVA results were used as a mask, and *post-hoc* analyzes within the areas were performed to investigate pairwise between-group differences (GRF-corrected *p* < 0.001, cluster level *p* < 0.05, cluster > 50).

Correlation analysis of zFC values of the LSM region showing significant differences was performed in the DPN and ND groups. Subsequently, significant partial correlations (Bonferroni corrected, *p* < 0.05) were evaluated between the zFC values and clinical variables, with age, sex, and years of education as covariates.

## Results

### Clinical and neuropsychological data

Three participants were excluded from the final statistical analysis; one patient with DPN was excluded because of excessive motion, and two participants (one patient each from the DPN and ND groups) were excluded because they showed WMH scores > 2. Thus, 83 patients with T2DM (44 and 39 patients in the DPN and ND groups) and 43 HCs were enrolled in the study. According to previous studies to evaluate the stability of neuroimaging (33, 34), in this study, the power of the current sample size was 0.80 and the effect size was 0.75, which indicated that the results of this study were reliable. The demographic, clinical, and neuropsychological data of the three groups are presented in **Table 1**. The three groups showed no significant differences in age, sex, education level, blood pressure, TG, TC, LDL-C, HDL-C, serum urea, and serum creatinine concentrations, and CTT-1/2, MMSE, CDT, RAVLT immediate/delayed, and SDMT scores (all *p* > 0.05). However, MoCA scores were lower in T2DM patients than in HCs (*p* > 0.05). As expected, FBG, PBG, and HbA1c levels were significantly higher in both groups of T2DM patients than in HCs (all *p* < 0.001), but these variables did not differ between the DPN and ND groups (*p* values were 0.38, 0.997 and 0.727 respectively).

**Table 1:** Demographic, clinical, and neuropsychological characteristics of the participants.

Variable	DPN (n = 44)	ND (n = 39)	HC (n = 43)	*F*/χ2 value	*p*-value
Sex (male/female)	23/21	25/14	26/17	1.275	0.529^#^
Age (years)	55.61 ± 8.87	51.76 ± 8.92	53.69 ± 6.70	2.259	0.109
Education (years)	13.02 ± 2.65	14.25 ± 2.48	14.07 ± 3.06	2.484	0.088
Duration (years)	8.29 ± 6.47	8.46 ± 5.40	–	1.011	0.318^&^
Systolic BP (mmHg)	132.15 ± 17.80	124.33 ± 15.69	128.90 ± 16.18	2.299	0.105
Diastolic BP (mmHg)	82.97 ± 13.40	80.10 ± 9.94	84.16 ± 10.96	1.303	0.275
BMI (kg/m**^2^**)	24.80 ± 4.66	25.13 ± 3.33	23.14 ± 3.43	1.607	0.205
FBG (mmol/l)	8.13 ± 2.67	8.65 ± 3.22	3.96 ± 2.14	37.658	<0.001^ab^
PBG (mmol/l)	8.65 ± 6.02	8.74 ± 6.17	0.35 ± 1.27	38.533	<0.001^ab^
HbA1c (%)	7.76 ± 2.14	7.91 ± 2.08	5.09 ± 1.51	28.03	<0.001^ab^
TG (mmol/l)	2.03 ± 1.59	1.91 ± 1.41	1.67 ± 0.94	0.786	0.458
TC (mmol/l)	4.26 ± 1.35	4.13 ± 1.29	4.74 ± 1.46	2.241	0.111
LDL (mmol/l)	2.33 ± 0.76	2.47 ± 0.92	2.65 ± 0.97	1.413	0.247
HDL (mmol/l)	1.02 ± 0.27	1.05 ± 0.28	1.13 ± 0.32	1.705	0.186
Serum urea (mmol/l)	4.86± 1.94	4.56 ± 2.31	4.49 ± 1.95	0.384	0.682
Serum creatinine (µmol/l)	55.44 ± 21.84	52.48 ± 26.17	62.33 ± 23.86	1.828	0.165
MMSE	28.56 ± 1.51	28.33 ± 1.49	28.30 ± 1.85	0.299	0.742
MoCA	25.19 ± 2.76	25.26 ± 2.86	27.27 ± 1.14	10.838	<0.001^ab^
CDT	21.90 ± 8.37	24.15 ± 8.46	23.05 ± 6.68	0.841	0.434
CTT-1	83.06 ± 29.51	79.53 ± 41.13	71.07 ± 35.30	1.198	0.305
CTT-2	156.29 ± 16.70	153.61 ± 15.98	136.71 ± 13.92	0.468	0.628
RAVLT Immediate	43.51 ± 9.37	45.90 ± 9.71	41.35 ± 9.45	1.289	0.282
RAVLT Delay	8.65 ± 3.45	8.96 ± 2.85	7.76 ± 3.89	0.715	0.493
SDMT	36.62 ± 11.97	43.10 ± 12.47	43.82 ± 9.56	2.997	0.056

Data are presented as mean ± standard deviation or number (%) unless otherwise indicated. DPN, patients with type 2 diabetes mellitus and diabetic peripheral neuropathy; ND, patients with type 2 diabetes mellitus without diabetic peripheral neuropathy; HC, healthy control; BMI, body mass index; FBG, fasting blood glucose; HbA1c, glycated hemoglobin; TG, triglyceride; TC, total cholesterol; LDL-C, low-density lipoprotein cholesterol; HDL-C, high-density lipoprotein cholesterol; MMSE, Mini-Mental State Examination; MoCA, Montreal Cognitive Assessment; CDT, Clock-Drawing Test; CTT-1, Color Trails Test part 1; CTT-2, Color Trails Test part 2; RAVLT, Rey Auditory Verbal Learning Test; SDMT, Symbol Digit Modalities Test. ^#^P for the χ^2^ test; ^&^P for the two-sample t-test; ^a^ *post-hoc* paired comparisons showing significant differences between DPN patients and HCs; ^b^ *post-hoc* paired comparisons showing significant differences between NPN patients and HCs; ^c^ *post-hoc* paired comparisons showing significant differences between DPN and NPN patients.

### Between-group differences in the FC of the LSM

**Figure 2:**
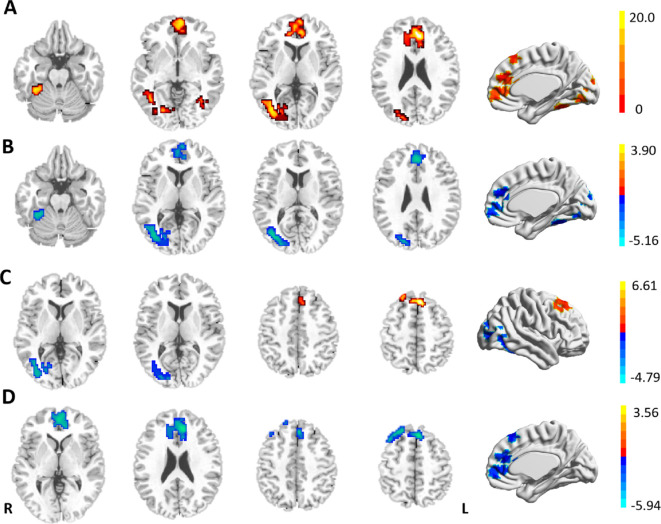
**(A)** Differences in the resting-state FCs of the LSM among the three groups. **(B)** Reduced FCs of the LSM in patients with DPN in comparison with HCs. **(C)** Brain regions showing differential FCs with the LSM in patients with DN and HCs. **(D)** Reduced FCs of the LSM in DPN patients in comparison with DN. GRF-corrected *p* < 0.001, cluster level *p* < 0.05, cluster >50. Warm (cold) color indicates significantly increased (decreased) FC.

The three groups showed significant differences in FC between the LSM and the right lateral occipitotemporal cortex (LOTC)/cerebellar lobule VI, the rostral prefrontal cortex (rPFC), the medial superior frontal gyrus (SFGmed), and the anterior cingulate gyrus (ACC) in the ANOVA (**Table 2**, **Figure 2A**). Between-group analysis revealed that in comparison with HCs, patients with DPN showed reduced FC of the LSM with the right cerebellar lobule VI, right LOTC, the rPFC, and the ACC (**Table 3**, **Figure 2B**); while patients in the ND group showed reduced FC of the LSM with the right LOTC and increased FC of the LSM with the SFGmed (**Table 3**, **Figure 2C**), In addition, in comparison with the ND group, patients with DPN showed reduced FC of the LSM with the ACC, SFGmed, and rPFC (**Table 3**, **Figure 2D**).

**Table 2:** Differences in the functional connectivity of the lower limb sensorimotor cortex among the three groups.

Brain region	X	Peak MNI Y	Z	Cluster size	BA	F-value
LOTC/cerebellum_VI _R	33	-81	6	393	19	4.6703
rPFC _B	0	51	0	199	10	4.8198
ACC_B	-6	39	24	179	32	5.11
SFGmed _B	-6	27	51	236	8	5.9903

LOTC, lateral occipitotemporal cortex; SFGmed, medial superior frontal gyrus; rPFC, rostral prefrontal cortex; ACC, anterior cingulate gyrus; B, bilateral; R, right; L, left.

**Table 3:** Between-group differences in the functional connectivity of the lower limb sensorimotor cortex.

	Brain regions	X	Peak MNI Y	Z	Cluster size	BA	F-value
**DPN-HC**	Cerebellum_VI _R	36	-45	-27	73	–	-4.7746
	rPFC _B	-6	60	-3	142	10	-4.7773
	LOTC _R	39	-75	6	175	19/37	-5.0963
	ACC _B	0	39	27	84	32	-5.1566
**ND-HC**	LOTC _R	42	-57	-6	201	19/37	-4.7854
	SFGmed _B	-6	27	51	121	8	6.6054
**DPN-DN**	rPFC _B	0	51	0	177	10	-5.2179
	ACC _B	-9	36	21	167	32	-5.6027
	SFGmed _B	-6	27	51	234	8	-5.9414

LOTC, lateral occipitotemporal cortex; SFGmed, medial superior frontal gyrus; rPFC, rostral prefrontal cortex; ACC, anterior cingulate gyrus; B, bilateral; R, right; L, left; DPN, patients with type 2 diabetes mellitus and diabetic peripheral neuropathy; ND, patients with type 2 diabetes mellitus without diabetic peripheral neuropathy; HC, healthy control.

### Correlation analysis

**Figure 3:**
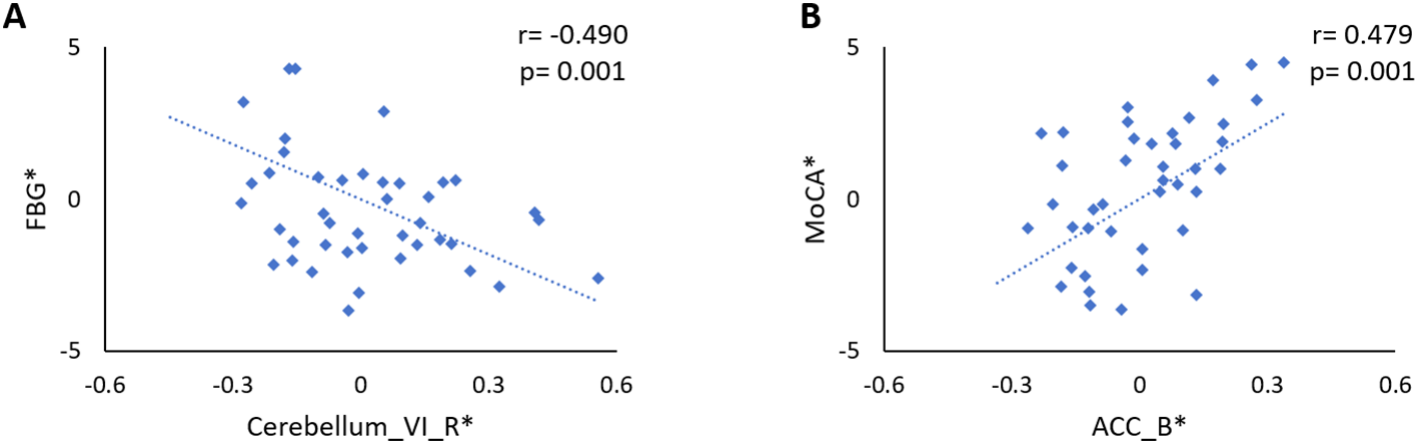
**(A)** Significant negative correlation between the FBG level and the FC between the LSM and the right cerebellar lobule VI in DPN patients (r = -0.490, *p* = 0.001). **(B)** The FC between the LSM and the right ACC was significantly correlated with the MoCA score in patients with DPN (r = 0.479, *p* = 0.001). The asterisk (*) indicated coordinate values controlling for the influence of age, sex, and years of education.

In patients with DPN, after controlling for age, sex, and BMI and Bonferroni correction for *P*, the FC between the LSM and the right cerebellar lobule VI was significantly correlated with the FBG level (r = -0.490, *p* = 0.001; **Figure 3A**) and the FC between the LSM and the right ACC was significantly correlated with the MoCA score (r = 0.479, *p* = 0.001; **Figure 3B**). Other FCs showing group differences were not associated with clinical/neuropsychological variables in patients with T2DM.

## Discussion

In this study, we found reduced FC between the LSM and the right LOTC in patients with different clinical stages of T2DM (with or without DPN). In addition, patients with DPN showed reduced FC between the LSM and several brain regions in the cerebellum and prefrontal lobe. These findings imply that the LSM shows abnormal functional coupling with the motor-related visual cortex in T2DM patients before the emergence of DPN. After the appearance of DPN, the LSM have influenced multiple motor cortex and cognition-related brain regions.

The LOTC is primarily located in areas 37 and 19 (35, 36), which is important for visually perceiving limb position and encoding visual information concerning motions (37, 38). This region is involved in the execution of limb movements (39) and is particularly sensitive to movement types such as foot lifting (40). Xin et al. (12) found reduced neuronal activity in the right middle temporal gyrus in patients without DPN, and an abnormal network hub has been identified in the LOTC of patients with DPN (41). These studies suggest that LOTC dysfunction may exist in patients with different clinical stages of T2DM. The LOTC is richly connected to several areas related to body movement control, including the precentral gyrus (35). The present study found that all patients with T2DM (ND and DPN group) showed reduced FC of the LSM with LOTC, indicating that the process of perception and motor control of the limbs may be impaired in T2DM patients. On the other hand, accurate stepping relies on the combined use of vision and motion control to determine the desired position of foot placement and subsequently control accurate movement of the lower limb. Previous study has suggested that DPN causes impaired motor control of the lower extremities and altered visual gaze strategies, resulting in decreased foot mobility (42), and our study provides imaging evidence for this.

Some scholars have previously suggested that the SFGmed (area 8) plays an important role in voluntary movement and maintenance of body balance (43). Chul et al. (44) found that metabolic activity in the SFGmed was associated with improved motor scores in treated patients with Parkinson disease. It has also been suggested that patients with cervical spondylosis may maintain relatively normal motor function by employing more motor brain regions (SM and superior frontal gyrus) (45). Considering the potential abnormalities of motor function that may exist in patients without DPN, the present study found that the increased FC of the LSM with the SFGmed in the ND group may be a compensatory mechanism for the central response to early motor dysfunction. In contrast, the decreased FC of the LSM with the SFGmed in patients with DPN may be related to sensory-motor impairments in the lower limbs of the patients. Motor control is influenced by peripheral feedback, and DPN can cause a loss of afferent input that provides information about the current state of the body and the ongoing target-directed movement, resulting in a loss of motor control feedback (46). A previous study also suggested that abnormal SFGmed function may be a key brain region in motor dysfunction in DPN patients (12).

Previous studies (47, 48) have found that learning motor skills can lead to cerebellar plastic changes within cerebellar lobules V and VI. Walking is a motor skill gradually acquired by humans after birth, and the control of learned actions us stored in the cerebellar model of movement (49). The present study found that the FC of the LSM with cerebellar lobule VI was reduced and negatively correlated with the FBG level in patients with DPN, which may indicate degradation of the patient’s previously learned motor skills wherein the intended movements deviated from the ideal state. Increased fasting glucose levels have been shown to be associated with reduced fine motor scores and are more pronounced in patients with T2DM (50). A recent study confirmed that selective disruption of cortico-cerebellar communication in mice, while not preventing the onset of movement, reduces the precision, accuracy, duration, or success of movement (51). Therefore, we hypothesized that hyperglycemia may trigger the degeneration of motor function by disrupting the SM-cerebellar lobule VI pathway, which may be the neural mechanism underlying lower limb movement disorders in patients with DPN.

Both the rPFC and the ACC are closely related to attentional function, with the rPFC being responsible for control of sustained attention (52), and the ACC being involved in preparatory attention (53). Motor skills are known to change attentional demands, and divided attention impairs immediate motor performance (54). Previous study (55) has shown activation of SM areas and the rPFC during one-handed grip-strength tests, while rPFC activation has been shown to increase with task intensity, potentially reflecting the greater attentional demands required to control motor output at high intensities. In addition, the FC between the SM and the ACC has been shown to correlate significantly with the clinical assessment of motor function in children with developmental coordination disorder (56). Therefore, we speculate that the reduced FC between the LSM and these two brain regions in patients with DPN may imply that peripheral nerve damage affects motor-related attentional function. Interestingly, a trend toward statistically significant differences was observed in the SDMT test (*p* = 0.056) among the many cognitive domain scores. The SDMT relies heavily on eye-hand-motor coordination and requires the motor-related attention (57, 58). The patients with DPN usually present with subclinical neurological changes in the hands and/or fingers and may cause reduced eye-hand-motor coordination, which seems to supports our hypothesis to some extent. In addition, the MoCA scores of patients with DPN in the present study were significantly lower than those of HCs and positively correlated with the FC between the LSM and the ACC, suggesting that dysfunctional connectivity in the SM may contribute to patients’ impaired cognitive function.

## Limitations

This study had some limitations. First, although the present study could only indirectly reflect the effect of DPN on brain function in patients, and a simple and easy way to directly reveal the link between DPN and brain damage may be difficult to find. Second, the current study did not grade the severity of peripheral neuropathy to analyze the exact relationship between the disordered FC pathway and disease severity. In the future, we will explore and identify the core pathways of DPN-related brain damage. Third, the changes in the spinal cord structure of the patients were not explored to characterize the effects of DPN on the CNS in a comprehensive and systematic manner. Fourth, considering that other microvascular complications (retinopathy and nephropathy) may affect central sensorimotor and cognitive functions (59, 60), patients with other microvascular complications were excluded from this study, which makes our results lack generalizability to reflect the entire course of the disease, but this also improves the reliability of the results of this study.

## Conclusion

Based on the projection area of lower limb sensorimotor information in the brain, this study is the first to depict the patterns of FC between the LSM and the whole brain in patients with different clinical stages of T2DM, indirectly revealing the potential impact of peripheral neuropathy on the central nervous system. The results suggested that patients showing T2DM without DPN may have abnormal visual perceptual functions related to motion. Importantly, DPN may influence the brain regions that maintain motion and motor control, and this effect is not limited to motor function. This study suggests the need for endocrinologists to conduct a comprehensive assessment of motor-related cognitive function in patients with DPN and to tailor treatment, which may help to slow the progression of brain damage and improve patients’ quality of life.

## Data Availability

The raw data supporting the conclusions of this article will be made available by the authors, without undue reservation.
